# Impact of Distribution and Network Flushing on the Drinking Water Microbiome

**DOI:** 10.3389/fmicb.2018.02205

**Published:** 2018-09-19

**Authors:** Joline El-Chakhtoura, Pascal E. Saikaly, Mark C. M. van Loosdrecht, Johannes S. Vrouwenvelder

**Affiliations:** ^1^Department of Biotechnology, Faculty of Applied Sciences, Delft University of Technology, Delft, Netherlands; ^2^Water Desalination and Reuse Center, Division of Biological and Environmental Science and Engineering, King Abdullah University of Science and Technology, Jeddah, Saudi Arabia

**Keywords:** 16S rRNA gene pyrosequencing, biofilm, core microbiome, drinking water distribution system, microbial ecology, network flushing, residence time, water quality

## Abstract

We sampled the tap water of seven unique, full-scale drinking water distribution systems at different locations as well as the corresponding treatment plant effluents to evaluate the impact of distribution and the potential presence of a core drinking water microbiome. The water was also sampled during network flushing to examine its effect on the microbial ecology. While a core microbiome dominated by *Gammaproteobacteria* was found using 16S rRNA gene pyrosequencing, an increase in biomass was detected in the networks, especially during flushing. Water age did not significantly impact the microbiology. Irrespective of differences in treatment plants, tap water bacterial communities in the distinct networks converged and highly resembled the flushed water communities. Piping biofilm and sediment communities therefore largely determine the final tap water microbial quality, attenuating the impact of water source and treatment strategy and highlighting the fundamental role of local physicochemical conditions and microbial processes within infrastructure micro-niches.

## Introduction

Drinking water is nowhere near a sterile environment; after leaving a treatment plant it travels long distances in large distribution networks that are home to hundreds or thousands of species of bacteria, archaea, viruses, fungi, and invertebrates (van der Wielen et al., 2009; Ingerson-Mahar and Reid, 2012). However, most of these organisms are benign; e.g., it is estimated that completely safe water contains 10^6^–10^8^ bacterial cells per liter (Hammes et al., 2008; Lautenschlager et al., 2010). Excessive microbial growth in DWDSs can become problematic though when it causes pipe corrosion (Lee et al., 1980; Beech and Sunner, 2004), nitrification (Lipponen et al., 2002; Regan et al., 2003), aesthetic (water taste, odor, and discoloration) (Hoehn, 1988; van der Kooij, 2000), and/or health (pathogen proliferation) (Emtiazi et al., 2004; Thomas et al., 2004) concerns. Waterborne illnesses continue to occur today even in developed countries that employ advanced water treatment technologies, where, e.g., in the United States 4–32 million cases are reported annually (Colford et al., 2006; Messner et al., 2006), and the primary cause of drinking water-associated disease outbreaks is *Legionella pneumophila* in building plumbing systems (Beer et al., 2015). Characterizing the microbial communities indigenous to DWDSs and understanding network micro-scale processes is essential for assessing contamination risks and optimizing engineering designs.

A drinking water distribution system is a dynamic ecological niche for microbes that may be influenced by selection, drift, dispersal, and speciation processes whereby the resultant microbial tap water quality reflects historical population dynamics within the community as it was transported through a complex network interacting with its surroundings (Vellend, 2010; El-Chakhtoura et al., 2015; Schroeder et al., 2015). Research on the drinking water microbiome with high-throughput sequencing methods is relatively nascent compared to, e.g., the human gut, ocean, or soil microbiomes (Bautista-de los Santos et al., 2016). While most studies have reported *Alpha-* and *Beta-Proteobacteria* as the dominant bacterial groups thriving under different network physico-chemical and operational conditions, bacterial community structure and diversity has varied between DWDS studies (Lautenschlager et al., 2013; Holinger et al., 2014; Liu et al., 2014; Pinto et al., 2014) and occasionally different findings have been reported in different DWDSs pertaining to the impact of treatment strategy, temporal, spatial, hydraulic, and abiotic factors on drinking water microbiota. Investigating multiple DWDSs simultaneously applying a consistent methodology provides an opportunity to explore the core drinking water microbiome (or lack thereof) while avoiding biases that may arise from various sampling, DNA extraction, PCR amplification and sequencing protocols used in different studies. In this study we sampled the water of seven unique, full-scale DWDSs at different locations as well as the corresponding WTP effluents in order to evaluate the impact of distribution and the potential presence of a baseline drinking water microbiome. We also sampled the water during flushing of the networks (routinely performed for cleaning purposes) to examine its effect on the water microbiology.

## Materials and Methods

### Sampling Scheme

The research was conducted on seven full-scale drinking water treatment plants in Netherlands and their corresponding distribution networks. Each plant (labeled a–g) is unique, treating surface or/and groundwater applying various treatment strategies, listed in **Supplementary Table S1**. All the plants distribute drinking water that does not contain disinfectant residual. The pipes at the outlet of each WTP are made of steel and were built in different years, from 1967 to 1992 (**Supplementary Table S2**). As for distribution network pipes, either PVC or AC is used at the locations where water samples were collected, and construction/renovation dates range from 1950 to 2009 (**Supplementary Table S2**), with inner diameters of over 50 mm. Throughout the distribution networks though the pipes have different characteristics and sundry materials including cast iron and polyethylene are used. Bulk water samples were collected from (i) the treatment plant outlet (WTP), the distribution network (ii) before (TAP), and (iii) during (FLUSH) flushing. The network water samples (TAP and FLUSH) were taken from different locations, representing short, middle, and long distance from the treatment plant. The sampling took place in September/October 2011. A total of 56 water samples were collected and analyzed. For the WTP, two samples were collected from each outlet, a week apart, and the two samples were pooled as one in subsequent microbial analysis. As for the distribution networks (TAP and FLUSH), three samples were collected from each network at different locations (short, middle, and long distance) with the average hydraulic residence time estimated by the local water utilities to be 12, 23 and 41 h, respectively. These three samples were collected on the same day, on different days for the different plants (Sept. 14 for plant a, Oct. 10 for plant b, Oct. 10 for plant c, Sept. 7 for plant d, Oct. 6 for plant e, Oct. 12 for plant f, and Sept. 21 for plant g). TAP samples were collected from household taps. Unidirectional network flushing was conducted by applying high flow velocities (extra flow of 1.0 m s^−1^) as described by van Lieverloo et al. (2004) and samples were collected from fire hydrant faucets (on mains adjoining the sampled houses) during the flushing process (**Supplementary Figure S1**). Depending on the required subsequent analysis, samples were collected in specific, separate bottles as described by Prest et al. (2014). All samples were transported on ice to the laboratory, stored at 4°C and processed within 24 h. For 16S rRNA gene pyrosequencing, each 3 L sample was filtered through a 0.2 μm-pore-size Isopore membrane filter using sterile (autoclaved) filtration units (Millipore, Billerica, MA, United States) and the filters were stored at –20°C until processing.

### Physico-Chemical and Microbial Analysis

Each water sample was analyzed for temperature, turbidity, TOC, ATP, and HPCs. Temperature and turbidity were measured directly on site. TOC was measured according to a Dutch standard procedure (NEN-EN 1484). ATP analysis was carried out as described previously by Magic-Knezev and van der Kooij (2004). A pre-calibrated luminometer (Celsis Advance TM230) was used to measure the intensity of the emitted light. The detection limit of the method was 1 ng ATP per litre L^−1^. Unlike free ATP measurements, a nucleotide-releasing buffer step was added for total ATP analysis. Bacterial ATP concentrations were calculated by subtracting free ATP from total ATP concentrations. Free ATP values were below the detection limit in most samples. The HPC method was performed with yeast extract agar and the plates were incubated at 22°C for 3 days (Dutch procedure NEN-EN-ISO 6222).

### 16S rRNA Gene Pyrosequencing and Data Processing

Genomic DNA was extracted from the collected (filtered) biomass using the FastDNA SPIN Kit (MP Biomedicals, Santa Ana, CA, United States) according to the manufacturer’s instructions. Bacterial 16S rRNA genes were amplified with the bacteria-specific forward primer 515F (5′-LinkerA-Barcode-GTGYCAGCMGCCGCGGTA-3′) and reverse primer 909R (5′-LinkerB-CCCCGYCAATTCMTTTRAGT-3′). A single-step 28-cycle polymerase chain reaction (PCR) using the HotStarTaq Plus Master Mix Kit (Qiagen, Valencia, CA, United States) was performed for each DNA sample (triplicate reactions) under the following conditions: initial denaturation at 94°C for 3 min, followed by 28 cycles of 94°C for 30 s; 53°C for 40 s; and 72°C for 1 min; after which a final elongation step at 72°C for 5 min was performed. Following PCR, all amplicon products from different samples were mixed in equal concentrations and then purified using Agencourt AMPure beads (Agencourt Bioscience Corp., Beverly, MA, United States). Pyrosequencing was carried out at MR DNA Lab (Shallowater, TX, United States) on the Roche 454 FLX Titanium genome sequencer according to the manufacturer’s instructions. Sequence data was processed at MR DNA Lab. In summary, sequences were depleted of barcodes and primers, then sequences <150 bp were removed, as well as sequences with ambiguous base calls and with homopolymer runs exceeding 6 bp. Sequences were denoised, OTUs generated and chimeras removed. OTUs were defined by clustering at 3% sequence divergence (97% similarity). Final OTUs were taxonomically classified using BLASTn against a curated database derived from NCBI and Greengenes.

### Bioinformatic and Statistical Analysis

To study alpha-diversity Shannon–Weaver index and Chao1 richness estimator were computed for each sample using the R vegan package. Heatmaps showing relative taxonomic abundance were generated in the R Complex Heatmap package. Bar plots showing phylotype relative abundance were created using the R ggplot2 package. Venn diagrams showing unique and shared fraction of OTUs were created using the R VennDiagram package. MDS was performed with the Bray–Curtis matrix using the R statistical package to ordinate the OTU data (samples with similar community structure cluster together, taking into account the relative abundance of each OTU). ANOSIM was used to examine statistical significance between samples using the same Bray–Curtis distance matrix (vegan package within R). ANOSIM tests the null hypothesis that the average rank similarity between objects within a group is the same as the average rank similarity between objects between groups. It produces a test statistic (R) which can range from −1 to +1. Objects that are more dissimilar between groups than within groups will be indicated by an R statistic greater than 0. An R value of 0 indicates the null hypothesis is true. A level of significance (*p*-value) is also produced (Rees et al., 2004).

## Results and Discussion

### Change in Drinking Water Quality During Distribution and Flushing

**FIGURE 1:**
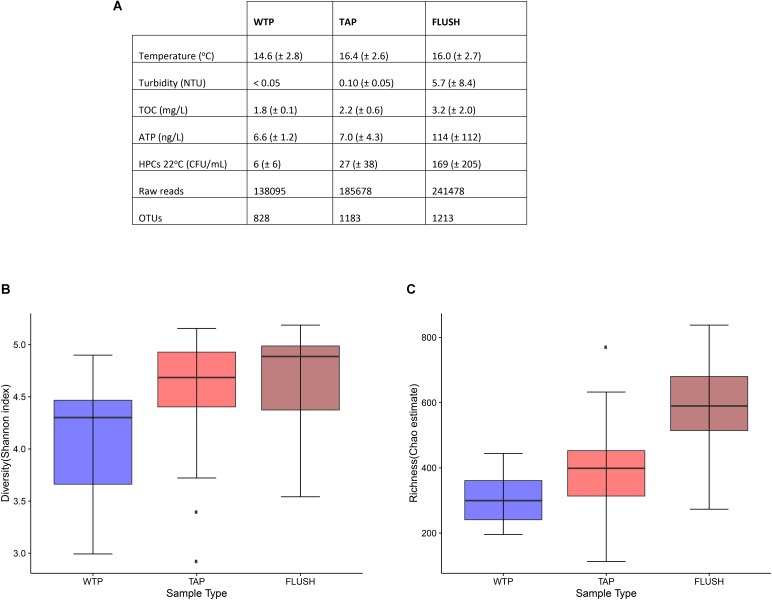
Water characteristics for treatment plant effluents (WTP), distribution network tap (TAP), and flushed (FLUSH) water. **(A)** Values represent the average (and standard deviation) of measurements taken from the seven plants and corresponding networks (14 WTP samples, 21 TAP samples, and 21 FLUSH samples). TOC, total organic carbon; ATP, Adenosine triphosphate; HPC, heterotrophic plate count; and OTU, operational taxonomic unit. **(B)** Shannon diversity and **(C)** Chao1 richness box plots: Displayed are IQRs or interquartile ranges (1^st^ and 3^rd^ quartiles; boxes), medians (horizontal lines in the boxes), minimum and maximum values within 1.5 IQR (whiskers below and above the boxes), and outliers (dark points beyond the whiskers).

The water physico-chemical and microbial parameters were measured at the production site and in the distribution networks at the taps before and during flushing (**Figure 1**). Some characteristics were affected by distribution and flushing. Most notable was the increase in bacterial richness and diversity, as indicated by HPCs, OTUs, and Shannon/Chao1 values, possibly indicating bacterial growth. Shannon diversity median values increased from 4.30 in the treatment plant effluent to 4.69 in the tap water and to 4.89 in the flushed water (**Figure 1B**). Chao1 richness median values increased from 299.56 in the WTP to 398.69 in the tap water and to 589.83 in the flushed water (**Figure 1C**). The largest range of variation was found in the community diversity (Shannon index) of the treatment plant effluents, likely due to the various water sources and treatment schemes used (**Supplementary Table S1**), which host or disseminate diverse bacterial communities in each unique system: surface or groundwater indigenous microbiota, species selected for with, e.g., the addition of different chemicals during the treatment process, innocuous bacteria that disperse from biological filter media, etc. As for potential bacterial growth in the DWDSs, the exact cause is unknown. For mesophilic bacteria the slightly warmer environment of the networks may have accelerated chemical and enzymatic reactions, triggering metabolic activity and growth (McCoy and VanBriesen, 2014). This is supported by the relative increase in ATP values. The alteration in pipe material from steel at all the WTP outlets to PVC or AC in the networks (and other various materials near the sample locations where the water had flowed) (**Supplementary Table S2**) may have been another factor. Different materials release different chemicals that act as substrates for some bacteria and as inhibitors for others. It has been reported that plastic substrates prompt biofilm growth due to the leaching of biodegradable compounds (Keevil, 2003). AC has also been linked with the growth of slime forming and heterotrophic aerobic bacteria (Wang and Cullimore, 2010). Aging of network pipes has also been correlated with changes in bacterial communities. It takes years for pipe-wall biofilms to mature, and old corroding pipes release particles that can serve as a nutrient source for microbes (Martiny et al., 2005; Ingerson-Mahar and Reid, 2012; Pinto et al., 2014). Moreover, little is known about how water microbiota responds to the insertion of new (disinfected but unsterile) pipes during renovation periods.

Bacteria in DWDSs grow in different microenvironments: the bulk water, biofilm attached to the inner pipe surface, loose deposits accumulated at the bottom of the pipe, and suspended solids transported through the mains (Liu et al., 2013). The contribution of the last two phases to total biomass diversity and activity is often overlooked and was found to be highest compared to the other phases (Liu et al., 2014), with, e.g., more than 90 bacterial cells per particle found in Liu et al. (2016). These four phases are dynamic and interchangeable under certain conditions. It is possible that during water distribution the increase in microbial parameter values was not due to bacterial growth but rather due to biofilm sloughing or deposit resuspension due to changing consumption or hydraulic events, causing the flow of some taxa into the bulk water phase. Similarly, during network flushing, high flow rates re-suspended deposits and exerted shear forces on the biofilm, causing attached and trapped bacteria (as well as any other non-sessile microorganisms) to (partially) dislodge and mobilize into the transported water. This phenomenon is supported by the significant increase in ATP, turbidity, and alpha diversity of the flushed water (**Figure 1**). What is uncertain is whether biofilms regenerate after this cleaning routine, and whether denser, more resistant communities evolve as an adaptation to turbulent flow conditions (Rochex et al., 2008; Abe et al., 2012).

### Bacterial Community Composition and Structure

**FIGURE 2:**
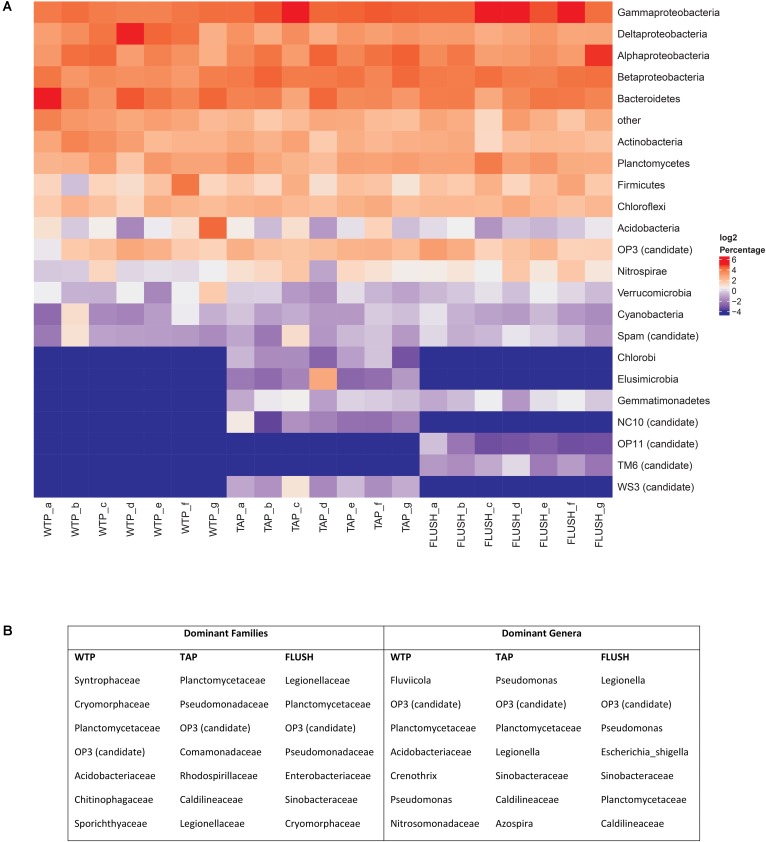
Bacterial groups in samples collected from seven water treatment plant (WTP) outlets and corresponding distribution network taps before (TAP) and during flushing (FLUSH). **(A)** Heatmap of bacterial phyla and *Proteobacteria* classes. Letters a–g represent the different WTPs as per **Supplementary Table S1**. Bacteria with a relative abundance below 1% across all samples were grouped together under the “other” category. **(B)** Top seven dominant bacterial families and genera detected in all WTPs, all TAP, or all FLUSH samples, listed in decreasing order of contribution to total reads.

Pyrosequencing revealed that the drinking water treatment plant effluents harbored a diverse bacterial community varying in structure at the different plants (**Figure 2A** and **Supplementary Figure S2**). The 12 detected phyla (including 2 candidate phyla) and their average relative abundance were: *Proteobacteria* (46.9 ± 5.6 %.), *Bacteroidetes* (15.2 ± 5.6 %), *Actinobacteria* (6.7 ± 2.7 %), *Planctomycetes* (5.8 ± 1.7 %), *Firmicutes* (3.9 ± 4.5 %), *Chloroflexi* (3.9 ± 1.0 %), *Acidobacteria* (3.5 ± 5.7 %), OP3 (3.4 ± 1.5 %), *Nitrospirae* (1.2 ± 0.5 %), *Verrucomicrobia* (1.2 ± 0.9 %), *Cyanobacteria* (0.7 ± 0.7 %), and Spam (0.6 ± 0.6 %). *Proteobacteria* classes were detected in the following order of decreasing abundance: *Gammaproteobacteria* (13.4 ± 1.6 %), *Deltaproteobacteria* (12.9 ± 6.3 %), *Alphaproteobacteria* (10.4 ± 4.3 %), and *Betaproteobacteria* (10.1 ± 2.2 %). In the distribution networks the bacterial community composition and structure changed (**Figure 2** and **Supplementary Figure S2**). Most prominent was the change in the dominant phylum subclass structure to: *Gammaproteobacteria* (18.1 ± 7.0 %), *Betaproteobacteria* (14.2 ± 3.7 %), *Alphaproteobacteria* (13.9 ± 6.6 %), and *Deltaproteobacteria* (7.3 ± 2.7 %), and the detection of five new phyla: *Elusimicrobia*, *Gemmatimonadetes*, *Chlorobi*, WS3, and NC10 candidate phyla. During network flushing 2 new candidate phyla were detected (TM6 and OP11) and the remaining community structure was similar to the bulk water TAP samples, particularly with respect to the *Proteobacteria* class structure and the dominant genera (**Figure 2** and **Supplementary Figure S2**).

*Gammaproteobacteria* have not been reported previously as the dominant *Proteobacteria* class in drinking water. This group has been found in abundance in piping biofilms and in loose deposits (Douterelo et al., 2013; Liu et al., 2014), indicating they may have migrated into the water from these two environments. *Betaproteobacteria* also tend to thrive in biofilms (Manz et al., 1999; Araya et al., 2003), suggesting their increased abundance during distribution may have been due to pipe biofilm sloughing. *Betaproteobacteria* have also been found more frequently in DWDSs lacking disinfectant residuals (Emtiazi et al., 2004; Lautenschlager et al., 2013) while other studies have found them to be significantly correlated with *Gammaproteobacteria* abundance and not with disinfectant residuals (McCoy and VanBriesen, 2014). The other detected phyla have been reported in other DWDSs (in the presence and absence of disinfectant residuals), in varying proportions. The relatively high diversity could denote a metabolically versatile community capable of adapting to changes in environmental or hydraulic conditions during water distribution. The exact cause behind the appearance of new phyla in the DWDSs is unknown, but likely due to interchange between the different network microenvironments described earlier. *Elusimicrobia* are anaerobes regularly encountered in termite hindguts (Herlemann et al., 2009). *Gemmatimonadetes* are abundant members of soil and sediment communities (DeBruyn et al., 2011), and *Chlorobi* comprise green sulfur bacteria closely related to *Bacteroidetes* (Hiras et al., 2016). WS3 is an anaerobic fermentative candidate phylum found in various terrestrial, aquatic, and marine ecosystems (Youssef et al., 2015) while NC10 constitute denitrifying methanotrophs (He et al., 2016). OP11 is a ubiquitous, yet poorly understood division (Youssef et al., 2011) and TM6 is dependent on eukaryotic hosts for its metabolic needs (Yeoh et al., 2016). Genera that were not detected in the WTP effluent but appeared during distribution are discussed further in the last section.

At family and genus level classification the seven most dominant groups are listed in **Figure 2B**. Most groups which were found to be dominant members of the distribution networks were originally present in the WTP effluents. *Pseudomonas* is a common DWDS member and increased from 2.18% at the WTPs to 4.27% in TAP samples and 3.79% in FLUSH samples. (The pathogenic species *Pseudomonas aeruginosa* was not detected.) *Legionella* increased from 0.69% at the WTPs to 2.62% in TAP samples and 6.66% in FLUSH samples. Although this group is common in building plumbing systems (particularly the biofilm component), its increase is noteworthy as some *Legionella* species are opportunistic pathogens. Pyrosequencing data is not suitable, however, for species-level classification as the sequences are too short for reliable identification and can only give us a certain percentage of homology to a species. Further testing is needed via, e.g., metagenomic approaches or designing primers that target specific species with qPCR to identify the bacteria at species level. Another dominant bacterial group detected in all the samples was the candidate division OP3. This group has been found in anoxic environments (Glöckner et al., 2010) and has also been detected as a dominant member of particle-associated bacteria in unchlorinated DWDSs (Liu et al., 2016).

### Core Microbiome Across Distributed and Flushed Water

**FIGURE 3:**
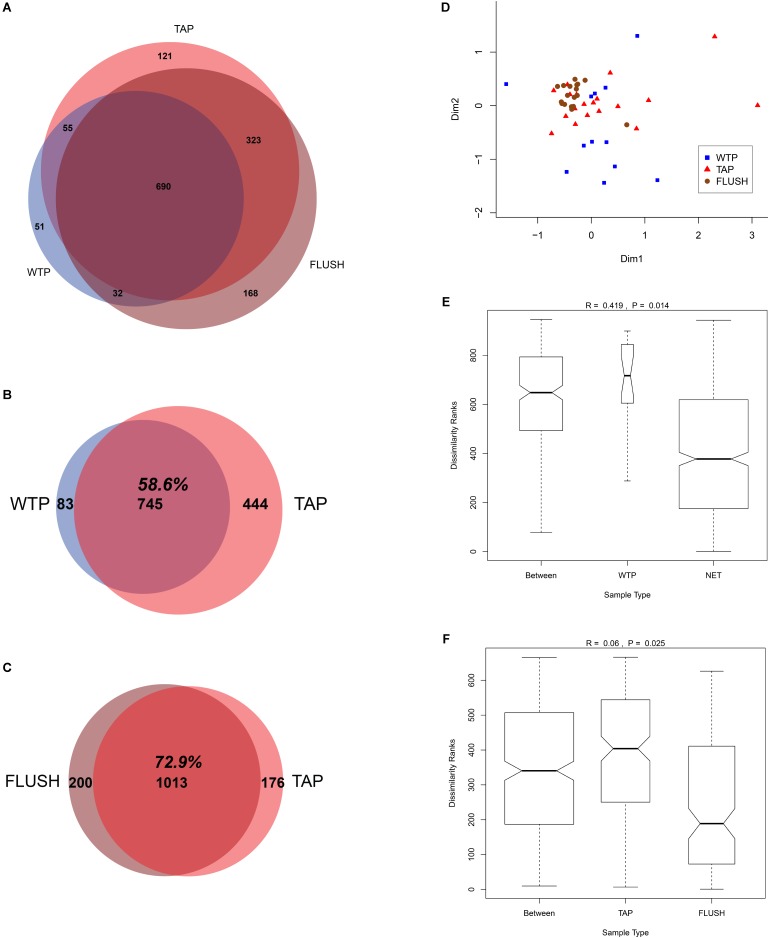
Core microbiome and statistical analysis of samples collected from water treatment plant outlets (WTP) and distribution network taps before (TAP) and during flushing (FLUSH). **(A–C)** Venn diagrams showing number of OTUs at each location and the shared fraction. **(D)** Multidimensional scaling (MDS) plot: each symbol represents an individual sample; the bigger the distance between samples, the bigger the difference in microbial community structure. **(E,F)** Analysis of similarity (ANOSIM) box plots showing within and between rank dissimilarities, with *R*- and *p*-values.

Despite the changes detected during distribution and flushing, a substantial fraction of the total OTUs (690/1440 or 47.9%) was shared between all the WTP, TAP and FLUSH samples (**Figure 3A**). This large fraction of common taxa constitutes the core microbiome for these drinking water systems, resilient to the stressful and oligotrophic conditions of the distribution network environment. The 14 WTP samples shared 82 bacterial genera and 83 families in common; the 21 DWDS TAP samples shared 171 genera and 126 families in common; and the 21 DWDS FLUSH samples shared 279 genera and 165 families. Comparing the 7 distribution networks to the seven treatment plants, 745 OTUs (58.6% of the total) were found at both sites (**Figure 3B**) while comparing the bacterial communities of the tap and flushed water (from all seven DWDSs), 1013 or 72.9% of the total OTUs (representing 198345 reads or 99.2% of the total normalized reads) were found in both types of water (**Figure 3C**). (Potential reasons behind the “disappearance” of 83 OTUs in the network include cell lysis by viruses and/or protozoa, the oligotrophic environment where some species have a competitive advantage over others, and migration from the water phase to the deposits or biofilm phase. For more details see El-Chakhtoura et al., 2015). Moreover, combining all the samples in one MDS plot showed that the WTP samples had a different bacterial community structure (for reasons discussed previously in the section “Change in drinking water quality during distribution and flushing”), with the samples collected from the plants with the same water source and treatment scheme (plants e, f, and g) grouped relatively adjacently (**Supplementary Figure S3**). The tap water samples from the various networks had a relatively similar community structure, and the flushed water samples clustered very closely (**Figure 3D** and **Supplementary Figure S3**), likely due to the more variable conditions found within households where tap water samples were collected. Additionally, the tap and flushed water samples clustered closely, separate from the WTP samples. These observations were confirmed statistically. Treatment plant and distribution network samples were statistically significantly different (*p* < 0.01; *R* = 0.419), with dissimilarity ranks highest within the WTP samples (**Figure 3E** and **Supplementary Figure S4**), while TAP and FLUSH samples were highly similar (*p* = 0.025; *R* = 0.06) and flushed water samples had the lowest dissimilarity rank (**Figure 3F** and **Supplementary Figure S4**).

This finding indicating the tap water microbiology highly resembles that of the flushed water–and to a lesser extent that of the treatment plant effluent–has not been reported before. Several studies have shown that the WTP and tap water communities are highly similar, with the treatment plant (e.g., communities colonizing biofilters) seeding and shaping the final tap water microbial community (Martiny et al., 2005; Pinto et al., 2012; Lautenschlager et al., 2013; Pinto et al., 2014). In a study that compared biofilm samples before and during network flushing (Douterelo et al., 2014), significant differences between the samples and also between different flushing sites were found. This study explored a single DWDS though containing residual disinfectant and the last recorded interventions within the network were 42 months previously, which could have allowed for more mature biofilms to grow (in our study network flushing is conducted at least once a year). Bulk water and biofilm phylotypes in DWDSs have been described generally as distinct communities (Martiny et al., 2005; Henne et al., 2012; Douterelo et al., 2014; Liu et al., 2014). The high similarity of the distributed and flushed water communities compared to the treated water in our study, as well as the elevated microbial load detected in the flushed water, reveal that the highly dynamic communities thriving within the biofilms and deposits had driven the microbial processes in the network and governed the change in the tap water microbiology during distribution. The biofilm and particle-associated communities therefore seem to act as a source from which migrants enter the bulk water community, shaping the resultant tap water quality. Furthermore, the convergence of the TAP and FLUSH bacterial communities from different networks is remarkable as it attenuates the impact of water source and treatment strategy and highlights the fundamental role of local physicochemical conditions and infrastructure quality. These findings most likely would not have applied in systems that do not deliver high-quality water in the first place, but seem to pertain to well-maintained DWDSs that are regularly cleaned (flushed) and subjected to similar operational (and environmental) conditions. Flushing could be a relatively rapid and simple method to evaluate tap water microbiology and levels of contamination risk.

### Similar Bacterial Community at Different Network Locations

**FIGURE 4:**
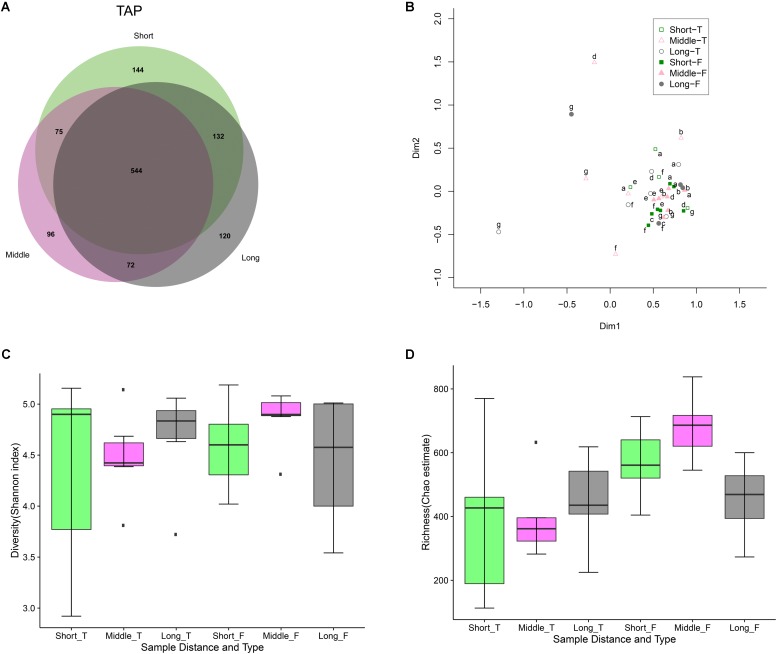
Tap and flushed water bacterial community variation with distance to treatment plant. **(A)** Venn diagram showing number of tap water OTUs at different distances and the shared fraction. **(B)** Multidimensional scaling (MDS) plot after eliminating two outliers from **Supplementary Figure S6**. Empty symbols represent TAP samples and filled symbols represent FLUSH samples. Letters represent the different water treatment plants as per **Supplementary Table S1**. **(C)** Shannon diversity and **(D)** Chao1 richness box plots for tap (T) and flushed (F) water: Displayed are IQRs or interquartile ranges (1^st^ and 3^rd^ quartiles; boxes), medians (horizontal lines in the boxes), minimum and maximum values within 1.5 IQR (whiskers below and above the boxes), and outliers (dark points beyond the whiskers).

The findings above inherently indicate that distance from the WTP (or water age) did not affect the drinking water microbiology. A large fraction of the total OTUs (544/1183 or 46%) (**Figure 4A**) and normalized sequence reads (285885/300000 or 95.3%) from all 7 distribution networks were shared between the different tap water locations (short, middle, and long distance to the plants). This was also the case for the flushed water, with 629/1213 or 51.9% of the total OTUs (**Supplementary Figure S5**) comprising 293845/300000 or 98% of the total reads shared between the different network locations. Combining all the distribution network tap and flushed water samples in one MDS plot revealed a similar bacterial community structure (with the exception of two outlier samples) (**Supplementary Figure S6**), and disregarding these two samples resulted in a similar clustering (**Figure 4B**) (with the FLUSH samples clustered more closely than the TAP samples). Examining the tap and flushed water samples separately in two MDS plots showed again no trend with distance, i.e., the samples were not grouped by network location (or residence time) (**Supplementary Figure S7**). ANOSIM confirmed that samples taken from different distance locations relative to the WTP were statistically not significantly different and in fact highly similar (**Supplementary Figure S8**). Drinking water samples collected from the locations nearest to the WTPs, however, had the highest number of OTUs and diversity/richness values (**Figures 4A,C,D**).

There have been mixed findings regarding the impact of water age or residence time on drinking water microbiology. In one chlorinated DWDS total bacterial cell concentrations varied with distance to the WTP (Nescerecka et al., 2014) while in other DWDSs containing residual disinfectant the bacterial community structure was found to be more variable in samples of low water age and ANOSIM showed water age had a significant effect on biofilm communities (Wang et al., 2014). In a study that examined DWDSs delivering water without residual disinfectant bacterial parameters were found to be stable with distance except one plant that showed high bacterial activity at proximal locations (van der Wielen and van der Kooij, 2010). In a chlorine-treated system that distributed water without residual disinfectant the water bacterial community was stable spatially (Henne et al., 2012), and in another disinfectant-free Dutch DWDS the water and biofilm communities were not affected by distance (Liu et al., 2014). Spatial effects have generally been reported to be less prominent than seasonal trends (McCoy and VanBriesen, 2014; Pinto et al., 2014), but it seems water chemistry shaped by the availability of residual disinfectant is a significant factor influencing the biological stability of DWDSs. The depletion of disinfectant residuals over time or distance is likely related to the change in water microbiology during distribution, while our study that analyzed 7 DWDSs substantiates that (well-treated) water transported without a residual disinfectant seems to be more biologically stable. As for relatively higher bacterial richness or activity at proximal locations in such systems that rely on nutrient limitation during treatment, a reason could be the availability of residual biodegradable organic matter in the areas close to the WTP, and/or due to bacterial removal by viruses and/or invertebrates in the areas further away from the plant. The high similarity of the flushed water bacterial communities at different network locations is expected as TAP and FLUSH microbiomes were highly similar. We would like to note that sediments and invertebrates were also sampled and analyzed and were found to be stable with distance in terms of volume, mass, and composition (data not shown).

### Bacteria Emerging During Drinking Water Distribution

**FIGURE 5:**
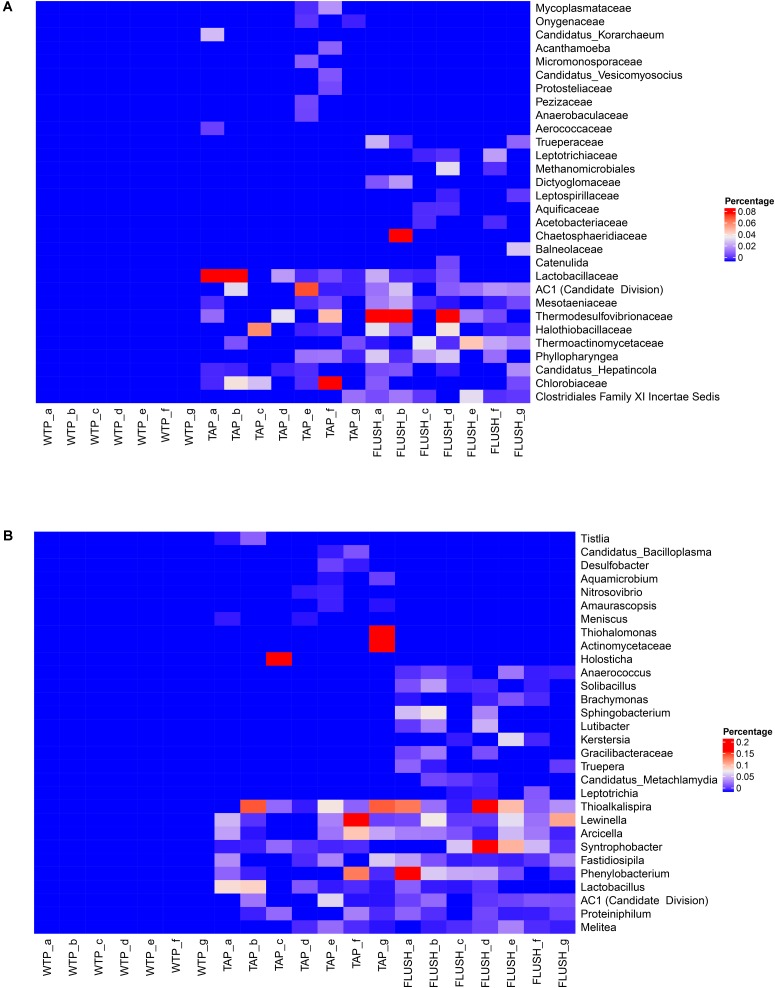
Bacterial groups absent in water treatment plant (WTP) effluents yet present in distribution network tap water (TAP) and/or flushed water (FLUSH). Heatmaps are shown at **(A)** family and **(B)** genus classification level. Letters represent the different WTPs as per **Supplementary Table S1**.

Bacterial families or genera that were not detected in the water leaving the treatment plants but appeared in distribution network (i) tap water, (ii) flushed water, or (iii) tap and flushed water are displayed in **Figure 5**, with the 10 most abundant groups listed for each of the three categories (percentage of total sequence counts did not exceed 0.2%, however, which is reflected in the Heatmap color coding). In total, while absent in WTP effluent samples, 15 families comprising 46 genera were found exclusively in tap water, 15 families or 71 genera were found solely in flushed water, and 76 families or 271 genera were found in both tap and flushed water samples. The most abundant genus detected only in the tap water (of plant g) was *Thiohalomonas*, a halophilic and facultatively anaerobic sulfur-oxidizing group belonging to *Gammaproteobacteria* and capable of complete denitrification (Sorokin et al., 2007). It is possible that sulfur compounds in the groundwater source of plant g were carried over to the distribution network allowing growth of these bacteria but it is unclear how the non-saline DWDS environment allowed them to thrive. As OTU sequence similarity to a *Thiohalomonas* strain was ∼92% as obtained by BLAST, the detected genus may instead have been a closely related sulfur-oxidizing group. The most abundant genus detected only in FLUSH samples (of plants a, b, and d) was *Sphingobacterium*, an aerobic chemoorganotrophic subgroup of *Bacteroidetes* having high concentrations of sphingophospholipids as cellular components. These bacteria exhibit sliding mobility (Yabuuchi et al., 1983), and this could have caused their migration from pipe biofilms into the flushed water at high flow rates. They have been found in cold-water plumbing systems in association with corroded pipes (Arens et al., 1995).

Bacteria that emerged during water distribution (not found at the treatment plant outlets) were more prone to be found in both the tap and flushed water samples (**Figure 5**). The most abundant genus (in terms of sequence counts) in this category was detected in all the DWDS samples (except the tap water from network a). It was *Thioalkalispira*, an alkaliphilic, chemolithoautotrophic, sulfur-oxidizing group belonging to *Gammaproteobacteria* and able to grow only under microaerophilic conditions (Sorokin et al., 2002). It is possible that cement lining in the water mains (mainly from AC pipes) provided an alkaline environment for these bacteria (Al-Mutaz et al., 1999; Deb et al., 2010) that potentially thrived within the biofilm on the cement surface, occasionally migrating into the bulk water. However, since OTU sequence similarity to a *Thioalkalispira* strain did not exceed ∼94%, further research is needed at species level to precisely identify these microorganisms. The second most abundant genus (detected in all the DWDS samples except the tap water from two networks) was *Lewinella*, of the *Bacteroidetes* phylum. They are aerobic, halotolerant chemoorganotrophs that exhibit gliding mobility and have been isolated from marine water and sediments as well as river epilithons (Sly et al., 1998; O’Sullivan et al., 2002), indicating they can thrive in different phases like bulk water, biofilm matrices and loose deposits. *Syntrophobacter* was also detected in most of the DWDS samples. These anaerobic *Deltaproteobacteria* grow syntrophically on propionate in the presence of hydrogen- and formate-utilizing bacteria or methanogens, and optimal growth is in fresh water at neutral pH (Harmsen et al., 1998). *Phenylobacterium* was another detected genus, a member of the *Alphaproteobacteria* class, comprising strict aerobes or facultative anaerobes that generally have a unique preference for phenyl moieties from heterocyclic compounds. They have been isolated from various environments including groundwater aquifers and drinking water reservoir sediments (Ginige et al., 2013; Cheng et al., 2014). *Arcicella* was another genus detected in the DWDS samples but not at the WTPs. They are aerobic, ring-forming bacteria (phylum *Bacteroidetes*) that grow in fresh water on a wide range of carbohydrates, and they have been isolated from tap water before (Kämpfer et al., 2009). DWDSs seem to sustain a unique, diverse ecosystem regardless of location or water origin.

## Prospects

The main findings of this study are: (i) A substantial, diverse core microbiome subsists in such DWDSs, dominated by *Gammaproteobacteria*; (ii) there is an increase in biomass in the distribution networks, especially during flushing, primarily due to pipe biofilm sloughing and loose deposit resuspension; (iii) residence time does not significantly impact the microbiology in (well-maintained) DWDSs transporting (well-treated) water without a residual disinfectant; (iv) piping biofilm and sediment communities largely determine the final tap water microbial quality, attenuating the impact of water source and treatment scheme; and (v) flushing is a relatively rapid, simple method to assess drinking water microbiology.

The finding that tap and flushed water microbiota are highly alike and even converge in multiple, distinct drinking water distribution networks has several implications. The water that is dispensed from our taps is not the same water that is produced by the treatment plant but rather a dynamic ecosystem driven by micro-niches within piping biofilms and loose deposits. The drinking water quality seems to reflect the “network quality”, shaped by the physical integrity of the system as well as local operational and natural conditions that select for specific microbes. This challenges efforts to predict, using mathematical models, downstream microbiology based on the composition of the community that leaves the treatment plant (Schroeder et al., 2015). Designing self-flushing networks with the optimal hydraulic regime and physical components to limit sedimentation and biofilm formation (and rejuvenation) is a challenging task but could help us “manipulate” the microbial ecology. A change in the water microbiology during distribution may not require control measures, however, when the hygienic (and aesthetic) quality of the water is not compromised, as the ultimate purpose of this built environment is to deliver safe water. Our understanding of drinking water microbial ecology is still limited and primarily based on DNA studies that do not tackle microbial function and processes. Moreover, due to the vital role of temperature, climate change is also bound to influence the microbial communities indigenous to DWDSs in ways we cannot prognosticate yet. This study shifts our focus from water treatment efficacy to distribution network (physical and hydraulic) quality and these findings need to be tested in DWDSs that apply a residual disinfectant.

## Data Availability

The pyrosequencing data generated and analyzed in this study is deposited in the NCBI SRA archive under accession number SRP136670.

## Author Contributions

All authors have contributed to this research paper. JSV set the research questions and experimental design, helped secure funding from Evides and KAUST, supervised the research, contributed to the interpretation of the results, and reviewed the manuscript. PS helped secure funding from KAUST, guided with the molecular biology approach and bioinformatic tools, and reviewed the manuscript. MvL helped secure funding from Evides, contributed to the interpretation of the results, and reviewed the manuscript. JC contributed to the design of the study, conducted the lab work, analyzed the data, interpreted the results, and wrote the article.

## Conflict of Interest Statement

The authors declare that the research was conducted in the absence of any commercial or financial relationships that could be construed as a potential conflict of interest.

## Abbreviations

AC: asbestos cement
ANOSIM: analysis of similarity
ATP: adenosine triphosphate
DWDS: drinking water distribution system
HPC: heterotrophic plate count
MDS: multidimensional scaling
OTU: operational taxonomic unit
PVC: polyvinyl chloride
TOC: total organic carbon
WTP: water treatment plant.
