# Supplementary material for: Impact of Distribution and Network Flushing on the Drinking Water Microbiome
Source: Front Microbiol. 2018 Sep 19;9:2205. doi: 10.3389/fmicb.2018.02205 (PMC6157312; doi:10.3389/fmicb.2018.02205)
Supplement: Supplementary file 1 [file Data_Sheet_1.pdf]

Table S1. Seven selected water treatment plants (WTPs) with corresponding water source and treatment scheme. coag= coagulation; floc= flocculation; fil= rapid dual layer filtration; GAC= granular activated carbon filtration; sed= sedimentation; O_3= ozonation; ms= microstrain; infil= infiltration; aer= aeration; soft= softening. The water is distributed without disinfectant residuals.

WTP	Source	Treatment
(a)	surface water	ms-coag-floc-sed-fil-UV-GAC-ClO_2
(b)	surface water	coag-floc-sed-O_3-fil-GAC-ClO_2
(c)	infiltrated surface water	ms-coag-fil-infil-aer-fil-GAC-UV
(d)	surface & groundwater	coag-floc-fil-fil-UV-GAC-ClO_2
(e)	deep groundwater	aer-fil-soft-fil
(f)	deep groundwater	aer-fil-aer-fil
(g)	deep groundwater	aer-fil-aer-fil

Table S2. Piping material and construction / renovation year for water treatment plants (WTPs) and corresponding distribution networks. Short, middle and long represent the distance between the WTP and the sampled network location. PVC= polyvinyl chloride; AC= asbestos cement. Throughout the distribution networks though the pipes have different characteristics and sundry materials including cast iron and polyethylene are used.

**WTP**	**Short**	**Middle**	**Long**
(a) steel 1967	PVC 1967	PVC 1968	PVC 1986
(b) steel 1976	PVC 1999	PVC 2000	PVC 1982
(c) steel 1977	PVC 1991	AC 1965	AC 1954
(d) steel 1967	PVC 1991	PVC 1987	PVC 1965
(e) steel 1992	PVC 2002	AC 1951	PVC 1979
(f) steel 1967	PVC 1988	PVC 1970	PVC 2009
(g) steel 1991	AC 1950	AC 1977	AC 1950

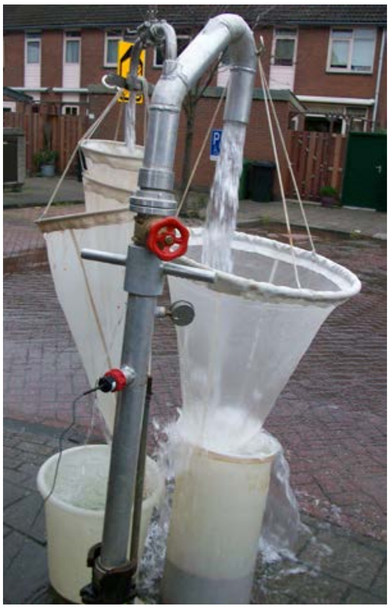

Fig. S1. Mains flushing. A fire hydrant is opened and using a high flow rate, water together with sloughed biofilm, loose deposits, and suspended solids are collected.

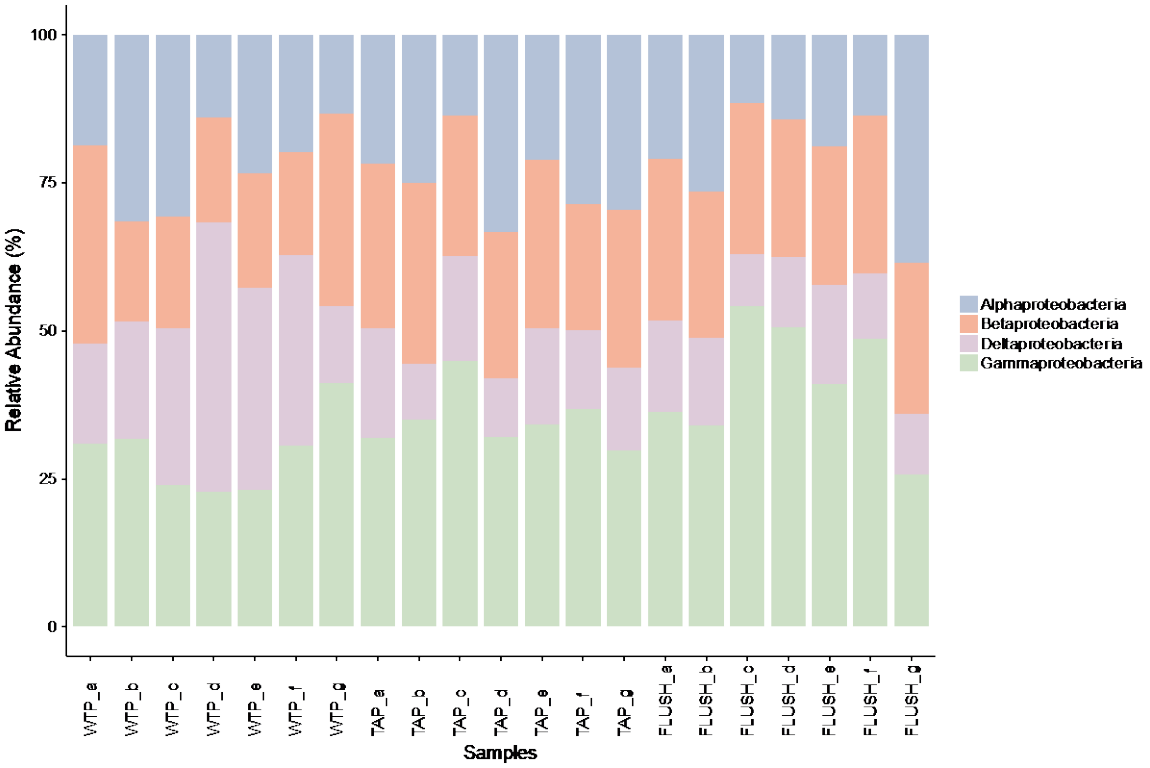

Fig. S2. Relative abundance bar plots of *Proteobacteria* classes of samples collected from 7 water treatment plant outlets (WTP) and corresponding distribution network taps before (TAP) and during flushing (FLUSH). Letters a to g represent the different water treatment plants as per Table S1.

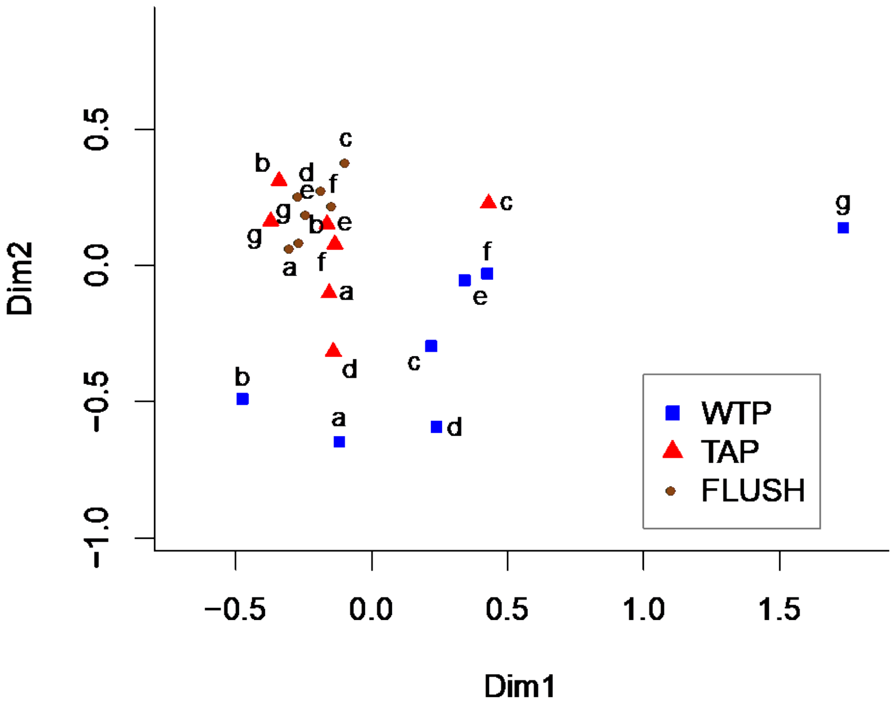

Fig. S3. Multidimensional scaling (MDS) plot of water treatment plant outlet samples (WTP) and distribution network tap samples before (TAP) and during flushing (FLUSH). Each symbol represents the pooling of 2 replicate samples for the WTPs and the pooling of 3 different distance samples for the TAP and FLUSH. The bigger the distance between samples, the bigger the difference in microbial community structure.

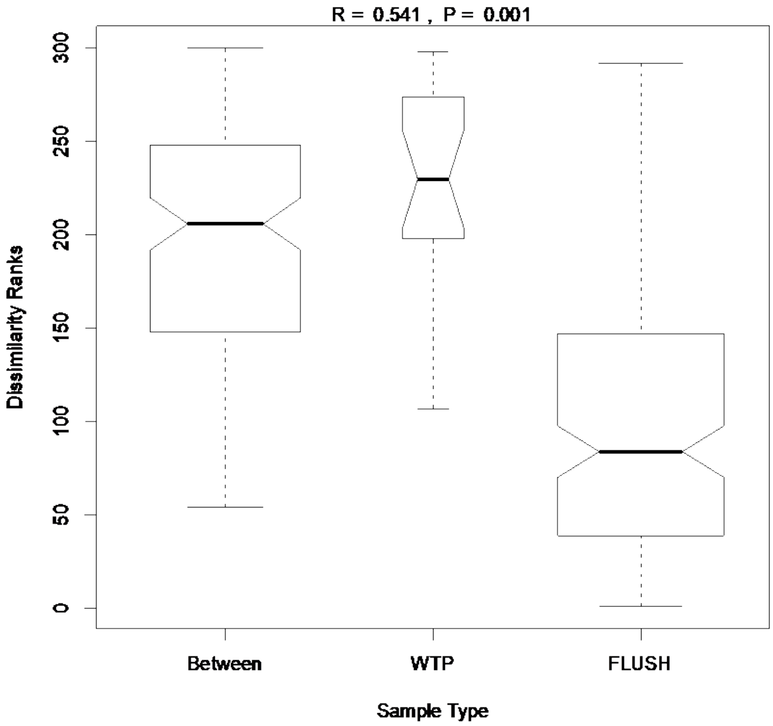

Fig. S4. Analysis of similarity (ANOSIM) box plot showing within and between rank dissimilarities for water treatment plant outlet samples (WTP) and distribution network flushed water samples (FLUSH), with R and p values.

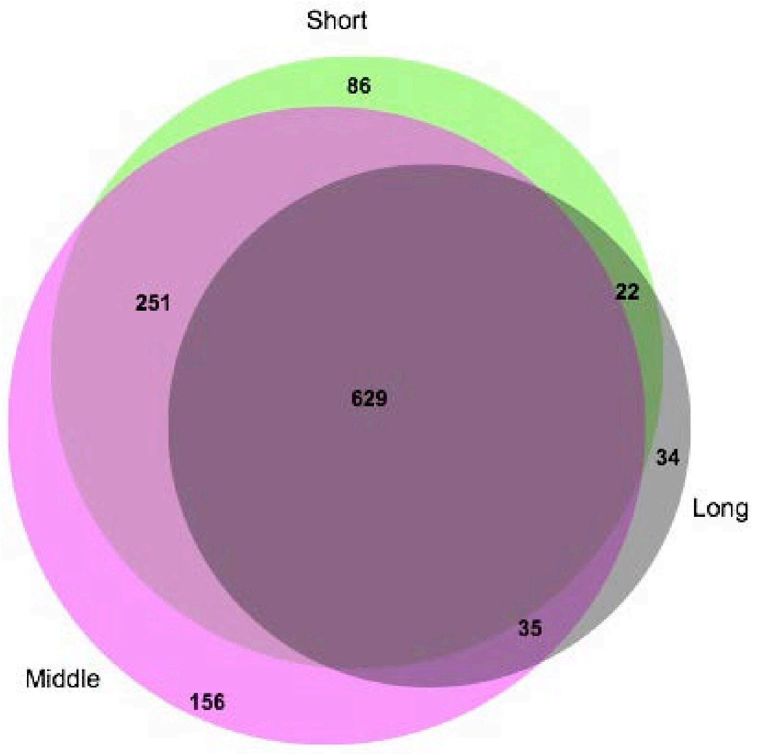

Fig. S5. Venn diagram showing number of flushed water OTUs at different network locations and the shared fraction.

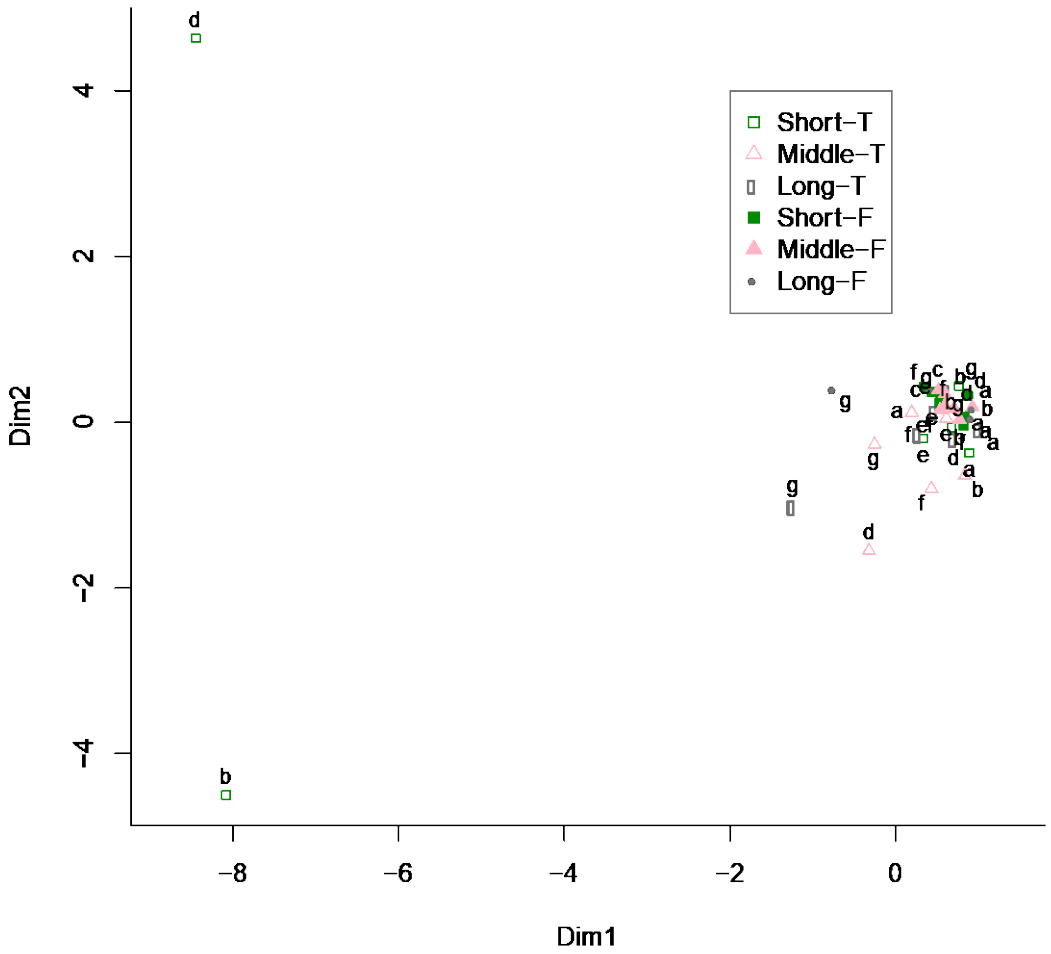

Fig. S6. Multidimensional scaling (MDS) plot of all distribution network samples. Empty symbols represent TAP samples and filled symbols represent FLUSH samples. Letters represent the different water treatment plants as per Table S1.

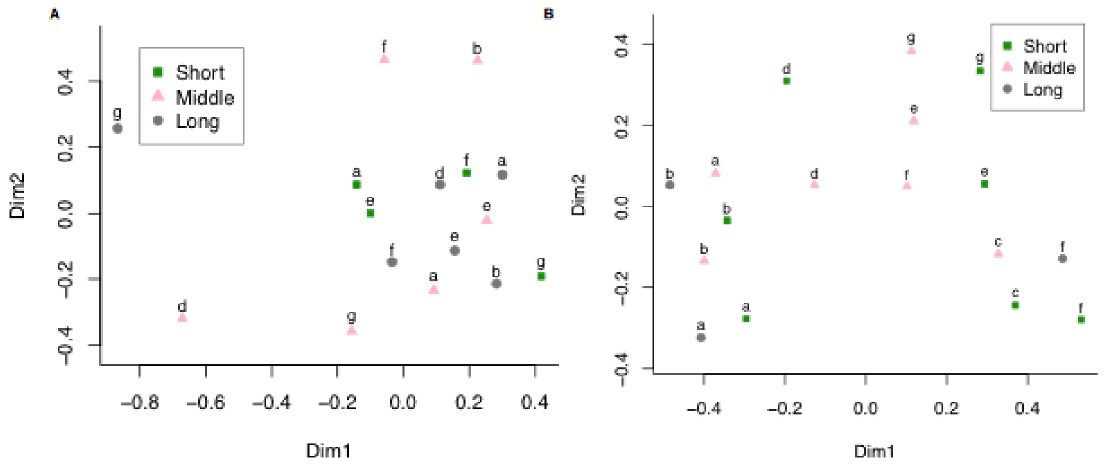

Fig. S7. Multidimensional scaling (MDS) plot break down of fig. S6 separating TAP (A) from FLUSH (B) samples. Short, middle and long represent the distance between the WTP and the sampled network location. Letters represent the different water treatment plants as per Table S1.

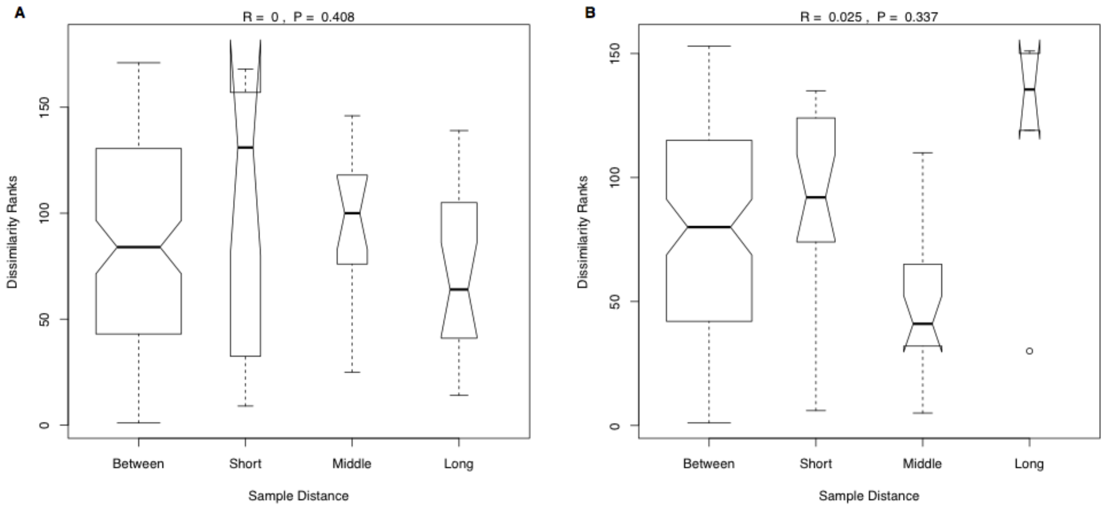

Fig. S8. Analysis of similarity (ANOSIM) box plots showing within and between rank dissimilarities for distribution network (A) tap and (B) flushed water samples, with corresponding R and p values. Short, middle and long represent the distance between the WTP and the sampled network location.
